# Protein kinase D-dependent CXCR4 down-regulation upon BCR triggering is linked to lymphadenopathy in chronic lymphocytic leukaemia

**DOI:** 10.18632/oncotarget.9031

**Published:** 2016-04-26

**Authors:** Stéphane Saint-Georges, Maude Quettier, Marouane Bouyaba, Stéphanie Le Coquil, Vanessa Laurienté, Lionel Guittat, Vincent Lévy, Florence Ajchenbaum-Cymbalista, Nadine Varin-Blank, Christine Le Roy, Dominique Ledoux

**Affiliations:** ^1^ INSERM U978, Bobigny, France; ^2^ Université Paris 13, Sorbonne Paris Cité, Labex “Inflamex”, Bobigny, France; ^3^ Assistance Publique-Hôpitaux de Paris, Hôpital Avicenne, Unité de Recherche Clinique, Bobigny, France; ^4^ Assistance Publique-Hôpitaux de Paris, Hôpital Avicenne, Service d'Hématologie Biologique, Bobigny, France

**Keywords:** CLL, lymphadenopathy, B-cell receptor, CXCR4/CXCR5, protein kinase D

## Abstract

In Chronic Lymphocytic Leukemia (CLL), infiltration of lymph nodes by leukemic cells is observed in patients with progressive disease and adverse outcome. We have previously demonstrated that B-cell receptor (BCR) engagement resulted in CXCR4 down-regulation in CLL cells, correlating with a shorter progression-free survival in patients. In this study, we show a simultaneous down-regulation of CXCR4, CXCR5 and CD62L upon BCR triggering. While concomitant CXCR4 and CXCR5 down-regulation involves PKDs, CD62L release relies on PKC activation. BCR engagement induces PI3K-δ-dependent phosphorylation of PKD2 and 3, which in turn phosphorylate CXCR4 Ser^324/325^. Moreover, upon BCR triggering, PKD phosphorylation levels correlate with the extent of membrane CXCR4 decrease. Inhibition of PKD activity restores membrane expression of CXCR4 and migration towards CXCL12 in BCR-responsive cells *in vitro*. In terms of pathophysiology, BCR-dependent CXCR4 down-regulation is observed in leukemic cells from patients with enlarged lymph nodes, irrespective of their IGHV mutational status. Taken together, our results demonstrate that PKD-mediated CXCR4 internalization induced by BCR engagement in B-CLL is associated with lymph node enlargement and suggest PKD as a potential druggable target for CLL therapeutics.

## INTRODUCTION

Chronic Lymphocytic Leukaemia (CLL) presents with a very heterogeneous clinical course from indolent to aggressive disease [1–5]. In spite of promising clinical results with recent signaling inhibitors, CLL remains incurable with standard therapy.

A current model proposes that mature CD5^+^/CD19^+^ CLL cells, which retain their sensitivity to external signals, cycle continuously between a quiescent state in the peripheral blood and a proliferative state in lymphoid organs [6]. Antigen-driven signals are involved in the progression of CLL [7–11], notably within the lymph node microenvironment [12, 13]. Binding of antigen to their receptor (BCR), initiates a set of signaling cascades leading to activation of protein kinases such as Spleen- (Syk) and Bruton -(Btk) tyrosine kinases or Phosphatidyl inositol 3-delta kinase (PI3K-δ). Targeting of these kinases [14, 15] interferes with the BCR/microenvironment crosstalk and allows peripheral cell redistribution [16]. Interestingly, inhibitors of Syk, Btk or PI3K-δ [17] also impair CLL cell migration induced by pro-survival chemokines such as CXCL12 upon binding to its cognate receptor CXCR4 [16]. Highly expressed at the surface of peripheral blood CLL cells due to its efficient recycling [18], CXCR4 mediates CLL cell chemotaxis and migration beneath and underneath CXCL12 secreting stromal cells [16]. In contrast, CXCR4 expression at the membrane of CLL cells is weak within the lymph node [13]. This is likely due to a ligand-dependent internalization of the receptor, which involves CXCR4 carboxy-terminal serine rich domain bound to various endocytic proteins [19–21]. Interestingly, in T cells, phorbol ester-induced CXCR4 endocytosis is sensitive to PKC inhibitors [19].

Initially identified as PKCμ, protein kinases D1 (PKD1) along with PKD2 and PKD3 form a new serine kinase subfamily [22–24]. The three proteins share two C1-domains, which bind diacylglycerol and phorbol esters (PMA) and an auto-inhibitory PH-domain [25]. Upon stimuli, both PKC-dependent and -independent activation of PKD members occur at serine residues [26, 27]. Phosphorylation of both Ser^744^ and Ser^748^ in the activation loop of the kinase domain is followed by trans- or auto-phosphorylation at Ser^916^ (a marker of PKD activation [28]) in the C-terminal region [29]. In adult mice, PKD2 is selectively expressed in murine lymphoid cells and controls their functions during adaptive immune responses [24]. Moreover, an *in situ* study revealed auto-phosphorylation of PKD2 in reactive lymph nodes and lymphoid tumors [30].

We have previously shown that *in vitro* BCR engagement induces plasma membrane CXCR4 decrease in CLL cells from progressive patients. Receptor internalization was related to decreased cellular chemotaxis towards CXCL12 gradient and correlated with shorter progression-free survival [10]. In this study, we addressed the molecular mechanisms underlying BCR-dependent CXCR4 down-regulation. We demonstrated that phosphorylation/activation of PKD in response to BCR stimulation, which involves PI3K-δ, is required for CXCR4-phosphorylation and its down-regulation. This regulatory pathway is functionally implicated in *ex-vivo* cell migration towards CXCL12 and correlated to the presence of lymph nodes in CLL patients.

## RESULTS

### PI3K and PKD2/3 activities mediate BCR-dependent CXCR4 down-regulation in CLL cells

**Figure 1:**
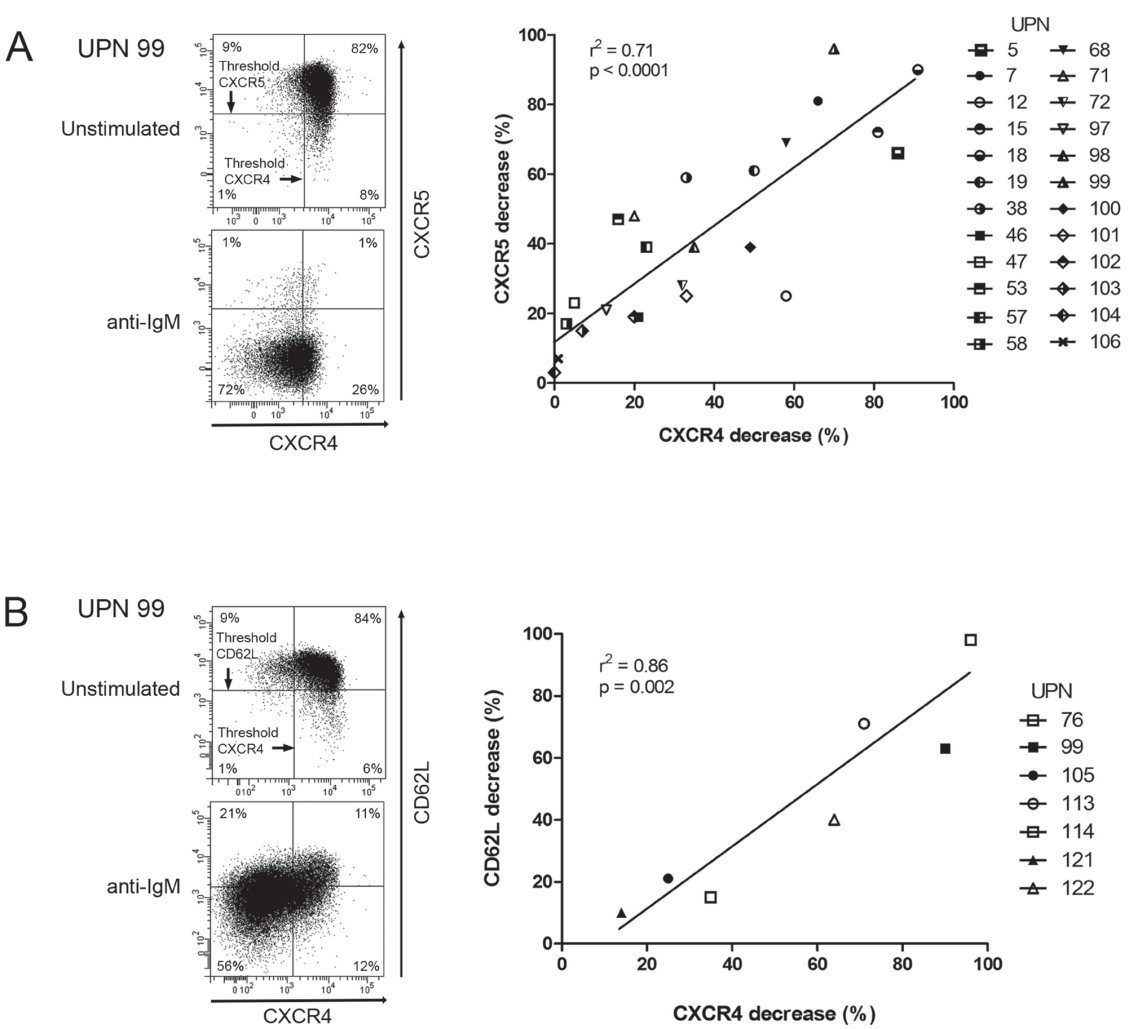
CXCR4, CXCR5 and CD62L are co-down-regulated in response to BCR triggering CLL cells were stimulated for 24 hours with anti-IgM antibodies. In CD19^+^/CD5^+^ cells, CXCR4 and CXCR5 **A.**, as well as CXCR4 and CD62L **B.** expressions were determined by flow cytometry (left panels) and percentages of CXCR4, CXCR5 and CD62L decreases were calculated and graphed (right panels).

We have previously demonstrated that the capacity for CLL B cells to down-regulate CXCR4 upon BCR engagement was correlated to shorter PFS [10]. We further strengthened this correlation on a new and larger cohort of 73 untreated CLL patients (Supplementary Figure S1 and Supplementary Table S1). Since enlarged lymph nodes, as CLL major proliferation sites, are an important clinical indicator of progression, we next investigated BCR-mediated CXCR4 downregulation capacity in patients presenting or not with lymphadenopathy (Table 1). Interestingly, all but one patients, with cells unable to downregulate CXCR4 (14/15), were stage A patients and did not harbor lymphadenopathy. In contrast, among cases with cells able to downregulate CXCR4, a majority had tumor burden and shorter time to first treatment (41/57). In lymph nodes, CXCR5 and CD62L are major players in homing, trafficking and adhesion of lymphocytes and in their tissue egress [31–35]. Strikingly, sustained antigenic stimulation of CLL cell samples promoted a similar CXCR5 downregulation and CD62L membrane release, suggesting the presence of a BCR responsive subclone (Figure 1).

**Table 1:** Extent of BCR-mediated CXCR4 down-regulation is correlated to lymphadenopathy from CLL patients

		BCR-mediated CXCR4 down-regulation	
		Low capacity (*n* = 15)	High capacity (*n* = 57)
Lymph node enlargement	Absence	14	16
	Presence	1	41

CLL cases (*n* = 72) were divided based on their cellular percentage of CXCR4 down-regulation in response to BCR trigering: Low capacity = CXCR4 decrease ≤ 5% and High capacity = CXCR4 decrease > 5%. Statistical analysis of the absence or presence of lymph nodes in both groups demonstrated that high BCR-mediated CXCR4 down-regulation was strongly linked to lymphadenopathy in CLL patients (‘Yates’ continuity corrected Chi2 test, *p* < 0.001).

**Figure 2:**
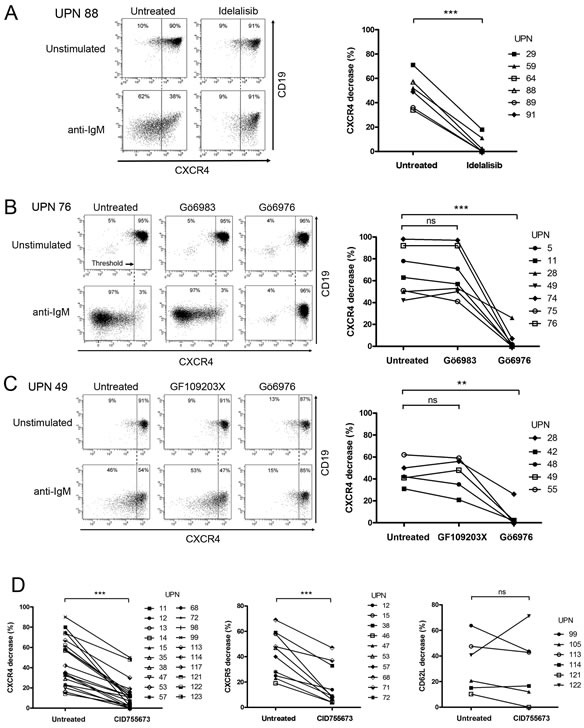
PI3K and PKD activities mediate BCR-dependent CXCR4/CXCR5 down-regulation but not CD62L release in CLL cells CLL cells were incubated for 24 hours in the presence (anti-IgM) or not (Unstimulated) of immobilized anti-IgM antibodies and in the presence or not (Untreated) of **A.** Idelalisib (50 μM), **B. C.** Gö6983 (1 μM), Gö6976 (1 μM), GF109203X (1 μM) or **D.** CID755673 (50 μM). CXCR4 and CD19 expressions were determined by flow cytometry. **A.**-**C.** Left panels show a representative sample for each treatment. Right panels depict the percentage of CXCR4 decrease upon stimulation that was calculated (cf. material and methods) and graphed from various CLL samples. **D.** shows BCR-mediated CXCR4 (left panel), CXCR5 (middle panel) and CD62L (right panel) decreases with (CID755673) or without (Untreated) PKD inhibitor. ** *p* < 0.001; *** *p* < 0.0001; *ns*, not significant.

In order to delineate the BCR effectors responsible for these modulations, we targeted early kinases of the pathway. As shown in Figure 2A and Supplementary Figure S2, inhibition of PI3Ks using a PI3K-δ specific inhibitor (Idelalisib) [14] or a pan-PI3K inhibitor (LY294002) [36], hindered BCR-dependent CXCR4 decrease in stimulated CLL cells. Importantly, treatment with the pan-PKC inhibitor Gö6983, which poorly inhibits PKD [37], or with the PKC inhibitor GF109203X that inhibits classical PKC isoforms, but not PKD [38], did not prevent CXCR4 decrease upon anti-IgM ligation [39]. Conversely, incubation of the cells with Gö6976, a selective inhibitor of classical PKC isoforms and purified PKD [40], blocked almost completely CXCR4 decrease (Figures 2B, 2C and Supplementary Figure S3). Then, dose response analysis with the potent and selective PKD inhibitor CID755673 [41], further assessed the functional involvement of PKDs in BCR-mediated CXCR4 decrease (Figure 2D left panel and Supplementary Figures S4 and S5A). Moreover, treatment with CID755673 blocked significantly BCR-mediated CXCR5 decrease (Figure 2D middle panel and Supplementary Figure S5B), demonstrating that PKDs also target CXCR5 clearance. In contrast, the membrane release of the CD62L selectin was not significantly altered by CID755673 treatment (Figure 2D right panel and Supplementary Figure S5C) but rather was inhibited by Gö6976 (Supplementary Figure S5D).

Altogether, these data demonstrate that membrane CXCR4/CXCR5 decrease in response to sustained BCR stimulation involves PKDs and that BCR-CD62L signaling cascade was rather controlled by PKCs.

### Involvement of a BCR/PI3K/PKD/CXCR4 phosphorylation axis

**Figure 3:**
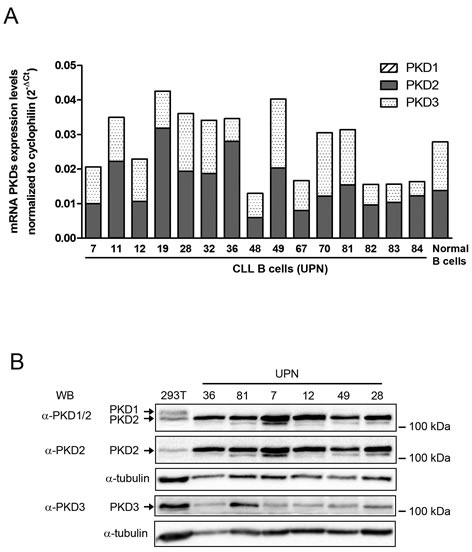
PKD2 and PKD3 are expressed in CLL cells **A.** PKD1, 2 and 3 mRNA expression levels were quantified by RT-qPCR in CLL and normal B cells, normalized to cyclophilin and graphed. **B.** Protein extracts from 293T and CLL cell samples were separated on SDS-PAGE and analyzed by immuno-blotting with the indicated antibodies; α-tubulin expression was used as a loading control.

**Figure 4:**
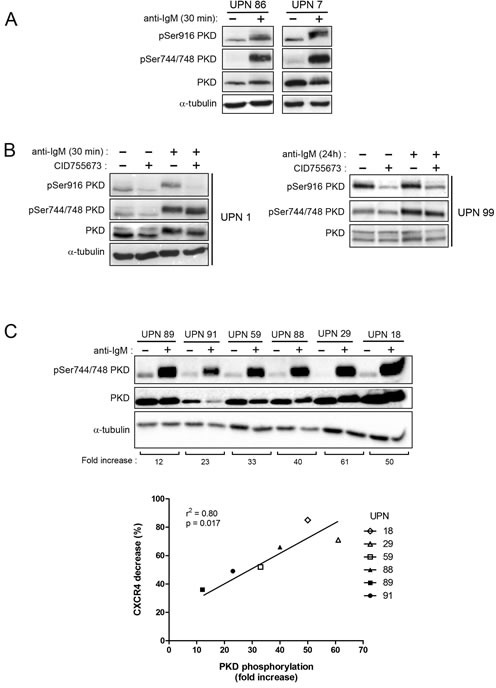
BCR engagement leads to specific PKD phosphorylation correlated with CXCR4 down-regulation in CLL responsive cells **A. B.** CLL cell samples were stimulated (+) or not (−) with anti-IgM for 30 minutes or 24 hours in the presence (+) or not (−) of 50 μM CID755673. Phospho-Ser^744/748^, phospho-Ser^916^ and total PKD (PKD1/2) contents were analyzed by western blotting using the indicated antibodies. **C.** CLL cell samples were stimulated (+) or not (−) with anti-IgM for 30 minutes and fold increase of phospho-Ser^744/748^ PKD (normalized to total PKD) were calculated for each UPN and graphed relative to BCR-dependent CXCR4 decrease (Supplementary Table S1).

Then, we investigated expression levels of the 3 PKD isoforms in CLL cells. Quantitative PCR showed that both malignant and normal B cells expressed PKD2 and PKD3 but not PKD1 mRNA (Figure 3A). Western blotting analysis of 6 CLL cell lysates using anti-PKD1/2 (recognizing both PKD1 and PKD2), anti-PKD2 and anti-PKD3 antibodies confirmed variable expression of PKD2 and PKD3 proteins and absence of detectable PKD1 as compared to 293T cells expressing the 3 isoforms [42] (Figure 3B). Since extracellular stimuli result in rapid PKD phosphorylation [23], we evaluated their phosphorylation status using either anti-phospho-Ser^744/748^ antibody (phosphorylation site present in all PKD isoforms) or anti-phospho-Ser^916^ antibody (site present in PKD1 and PKD2 only). Upon anti-IgM stimulation, phospho-Ser^744/748^ and phospho-Ser^916^-PKD were detectable already at 30 minutes and lasted for at least 24 hours (Figure 4A and 4B). Pharmacological inhibition of PI3Ks using LY294002 or the PI3K-δ specific Idelalisib substantially reduced PKD phospho-Ser^744/748^ level (Supplementary Figure S6), suggesting that PI3K-δ was an upstream kinase in CLL cells. In contrast, CID755673 weakly inhibited anti-IgM-dependent phosphorylation of Ser^744/748^, but strongly interfered with phosphorylation on Ser^916^ [41] (Figure 4B) indicating the specific blocking of PKD self-phosphorylation. Furthermore, quantification of BCR-dependent phospho-Ser^744/748^ PKD increase in 6 CLL cell samples significantly correlated with the decrease of surface CXCR4 observed upon anti-IgM-triggering (Figure 4C). Collectively, BCR triggering allows PI3K-δ−dependent PKD phosphorylation with the subsequent down-regulation of membrane CXCR4.

### BCR-unresponsive CLL cells are still PMA-responsive

**Figure 5:**
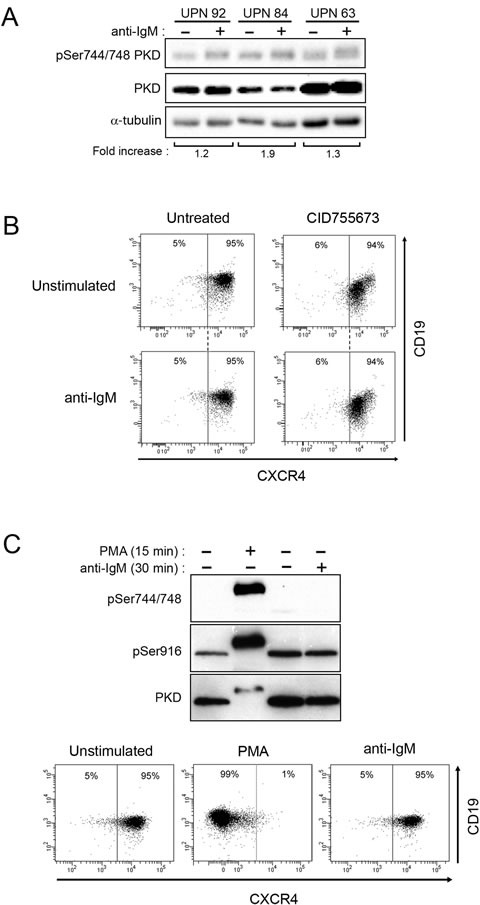
BCR-unresponsive cells respond to PMA treatment **A.** CLL cells were stimulated and analyzed as described in Fig. 4C. **B.** Upon stimulation or not with anti-IgM and with or not 24 h-treatment with CID755673, CXCR4 and CD19 expressions were determined by flow cytometry (UPN 50). **C.** CLL cells (UPN 4) were stimulated (+) or not (−) with anti-IgM or PMA (200 nM) for the indicated time. Phosphorylated (Ser^744/748^ and Ser^916^) and total PKD (PKD1/2) were analysed by western blotting (top). After 24 hours, CXCR4 and CD19 expressions were determined by flow cytometry (bottom).

Based on the extent of CXCR4 downregulation in response to sustained BCR stimulation, our cohort of 73 CLL patients was divided into 2 groups with one characterized by a high response (> 5%; *n* = 58) and another one with low response (≤ 5%, *n* = 15) (Supplementary Table S1). Analysis of the latters showed that phosphoSer^744/748^ PKD levels did not increase upon anti-IgM stimulation (Figure 5A); PKD or PKC inhibitors neither modified CXCR4 levels (Figure 5B and Supplementary Figure S7). All CLL samples, irrespective to their BCR-dependent CXCR4 down-regulation, remained responsive to PMA stimulation exhibiting increase of phospho-Ser^744/748^ and -Ser^916^ PKD levels, and strongly decreased their membrane CXCR4 (Figure 5C and Supplementary Figure S8), confirming their functional capacity.

### PKD is a BCR effector for CXCR4 phosphorylation and function

CXCR4 internalization upon CXCL12 binding involves phosphorylation of serine residues [20, 43]. Our *in silico* analysis revealed that Ser^325^ corresponds to a potential PKD phosphorylation consensus site (VSRGSS^325^); we investigated such a hypothesis.

**Figure 6:**
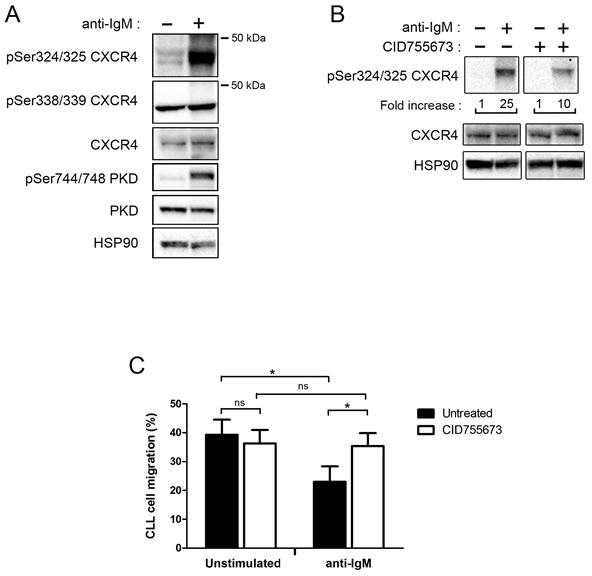
BCR engagement allows CXCR4 phosphorylation and reduces CLL migration toward CXCL12 *via* PKD **A.** CLL cells (UPN 96) were stimulated (+) or not (−) with anti-IgM antibodies for 30 minutes. Phospho-Ser^324/325^-, phospho-Ser^338/339^- and total-CXCR4, as well as p-Ser^744/748^- and total-PKD (PKD1/2) expression levels were analysed by western blotting using the indicated antibodies; anti-HSP90 antibody showed equal amount of protein in both conditions. **B.** CLL cells (UPN 88) were stimulated as described in A. and incubated in the presence (+) or not (−) of 50 μM CID755673. Fold increases of phospho-Ser^324/325^ CXCR4 are indicated below (both panels are issued from the same western blot). **C.** CLL cells were stimulated or not with anti-IgM and treated in the presence or not of 50 μM CID755673. After 24 hours, cells were subjected to a migration assay, counted by flow cytometry and graphed. Values represent the mean ± SEM of 4 independent experiments. * *p* < 0.05

Immunoblot analysis revealed a strong increase of phospho-Ser^324/325^ CXCR4 in stimulated CLL cells as compared to unstimulated ones, without any change of phospho-Ser^338/339^ or total CXCR4 levels in stimulated cells (Figure 6A). Moreover, treatment with CID755673 strongly impeded CXCR4 Ser^324/325^ phosphorylation (Figure 6B).

As expected [10], CXCR4 down-regulation in response to BCR engagement resulted in a significant reduction of the migration capacity of CLL cells towards CXCL12 *in vitro* (Figure 6C). Treatment with CID755673 restored the migration capacity to CXCL12 gradient (Figure 6C).

Taken together, our results argue for the phosphorylation of Ser^325^ upon BCR engagement *via* PKD leading to CXCR4 internalization and reduced mobility of the cells towards CXCL12.

### Extent of BCR-mediated CXCR4 is linked to tumor burden

**Figure 7:**
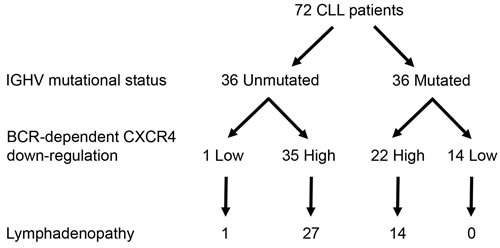
Extent of BCR-mediated CXCR4 down-regulation is related to lymph node enlargement from IGHV mutated CLL patients According to their IGHV mutational status (Unmutated and Mutated) and then on their BCR-mediated CXCR4 down-regulation (Low (≤5%) and High (> 5%)), the 72 CLL samples were divided into 4 sub-groups. Indicated numerals represent the number of patients in each sub-group. (‘Yates’ continuity corrected χ^2^ test: *p* < 0.001).

Finally, we challenged the BCR-induced CXCR4 downregulation in CLL cells with respect to their IGHV mutational status (Figure 7). According to their clinical progression, almost all cases (35/36) harboring unmutated IGHV exhibited a high BCR-mediated CXCR4 downregulation cell profile (mean ± SEM of CXCR4 decrease = 44.9% ± 20.3). In contrast, among the 36 IGHV mutated cases, two groups were identified, based on their cellular ability to downregulate CXCR4 in response to BCR stimulation. The first group including 14 IGHV mutated cases with low or none CXCR4 downregulation (mean ± SEM of CXCR4 decrease = 1.4% ± 2.0) remained in stage A. None of these patients had any lymph node progression in a median follow up of 8.4 years [Q1:4.4-Q3:9.9]. The second group, including 22 IGHV mutated cases, showed cells with a strong CXCR4 down-regulation (mean ± SEM of CXCR4 decrease = 49.2% ± 19.0). Among those, 14 patients (64%) developed clinical lymphadenopathy in a median follow up of 9.2 years [Q1:6.4-Q3:10.7]. These data indicated that CXCR4 downregulation reflects BCR signaling capacity, irrespective to the IGHV mutational status.

## DISCUSSION

We have previously shown that *ex-vivo* BCR engagement leads to a decrease of membrane CXCR4 expression in CLL cells from patients with unfavourable prognostic factors and at risk of disease progression [10]. In the present study, we further emphasized the BCR-dependent down-regulation of CXCR4 that was linked to tumor burden in both IGHV mutated and unmutated patients. We also deciphered the phosphorylation cascade leading to CXCR4 phosphorylation and to its subsequent endocytosis. This cascade consisted on a novel BCR-dependent pathway in which PI3K-δ phosphorylates PKD, which in turn phosphorylates CXCR4 at Ser^324/325^. We provided evidence that interrupting this pathway through inhibition of PKD rescued chemokine-driven cell migration that might contribute to CLL cell egress from lymph nodes *in vivo*.

PKD are important effectors in response to various stimuli in normal and pathological models [22–24]. Our data demonstrate that CLL cells co-express PKD2 and PKD3, but not PKD1. This pattern of expression is compatible to those described by immunohistochemistry in reactive lymphoid tissues and in neoplastic cells of lymphoid origin [30].

Both PKC-dependent and -independent pathways have been described in PKD activation [27, 28, 44–46]. In our study, we evidenced the upstream functional implication of PI3K-δ, rather than PKCs, in PKD-dependent CXCR4 and CXCR5 endocytoses upon antigen stimulation. Targeting PI3K-δ, which is abundantly expressed and constitutively activated in CLL cells [47], with Idelalisib has shown extensive efficacy for relapsed and refractory CLL with the drastic reduction of lymphadenopathy [14–16]. Besides uncovering a new regulation downstream of PI3K addressing CXCR4, we also showed that PKDs are involved in the endocytosis of an additional chemokine receptor, CXCR5. In contrast, shedding of CD62L rather implicates PKC in agreement with the described PKC/secretase/CD62L axis [48]. These data indicate that PKDs act as common signalling intermediates in BCR-dependent modulation of both CXCR4 and CXCR5 in CLL cells. It will be interesting to address whether these BCR signalling effectors are also involved in the regulation of several seven-transmembrane G protein-coupled receptors, including S1PR1 that controls lymphocytes egress from lymph nodes [49]. Interestingly, PKD involvement in BCR-mediated CXCR4/CXCR5 downregulation observed in our CLL samples appears to be different from the CXCL12/CXCR4 signaling pathway, which leads to CXCR4, but not CXCR5 and CD62L downregulation in the mouse model of TCL-1 CLL-like disease [50]. Based on the identification of CXCR4 as a partner of the BCR signalosome [51], a concomitant internalization between cell surface BCR, CXCR4 and CXCR5 upon antigen stimulation would not be surprising according to antigen targeted-regulation of these chemokine receptors.

In CLL cells, anti-IgM stimulation resulted in phosphorylation of Ser^744^ and/or Ser^748^ residues positioned in the activation loop of the PKD2 and PKD3 kinase domains and of Ser^916^ residue, a site of auto-phosphorylation present in the PKD2 isoform only [22–24, 28]. In absence of PKD1, CXCR4 down-regulation blocked by the CID755673, which interferes with PKD autophosphorylation at Ser^916^, pointed out phospho-PKD2 as the effector responsible for CXCR4 internalization.

Our experiments also indicate that in BCR-unresponsive cells, PKD is nevertheless functional. Whereas BCR engagement leads to only a weak phosphorylation of PKD and low or absence of CXCR4 internalization, treatment with the DAG analogue PMA overpassed this initial threshold response. This suggests that BCR-unresponsive CLL cells display a functional deficiency at the level of the BCR signalosome [11] rather than a defect of downstream effectors such as PKD.

**Figure 8:**
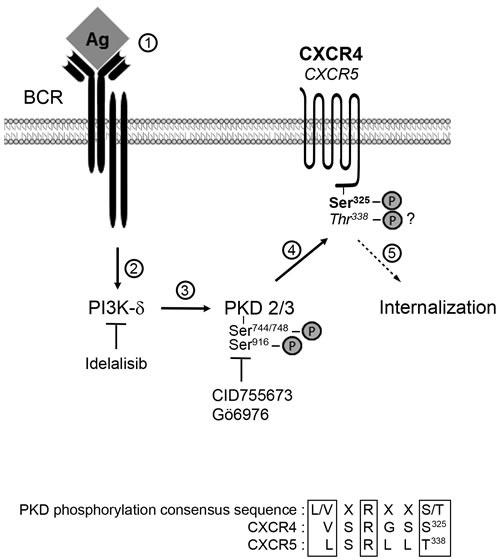
Cascade of events upon BCR trigerring in cases of lymphadenopathy Antigen binding to BCR (step 1) allows activation of PI3K-δ (step 2) and the subsequent increased phosphorylation of PKD2/3 on Ser^744/748^ and Ser^916^ (step 3). Activated PKD2/3 in turn phosphorylates CXCR4 Ser^324/325^ (and possibly CXCR5 Thr^338^) leading to GCPR internalization (step 5). Inhibitors of PI3K-δ and PKD2/3 are indicated. Consensus PKD phosphorylation sites altogether with the C-terminal sequence of CXCR4 and CXCR5 are aligned.

Our *in silico* analysis identified CXCR4 Ser^325^ and CXCR5 Thr^338^ as consensus sites for PKD phosphorylation (Figure 8). In line with this analysis, we demonstrated that CXCR4 Ser^324/325^ is a phosphorylated target of PKD upon BCR triggering in CLL cells. To our knowledge, no genetic variant of this residue has been described in CLL cells [52, 53]. A previous study described high levels of phospho-Ser^339^ CXCR4 in CLL cells compared to normal cells [54]. Our results not only confirmed this basal phosphorylation but also showed the absence of further activation upon BCR triggering. This suggests that phospho-Ser^338/339^ CXCR4 is not a target of BCR/PKD pathway in CLL cells but more probably a target of Btk/PKC upon CXCL12- or EGF-stimulations [50, 55]. Thus, our results in line with others propose that depending on the stimulus, different kinases are required for an appropriate CXCR4 regulation leading to alternative phosphorylation patterns [19–21, 43, 50, 54, 56, 57].

Based on the crucial roles of CXCR4 and CXCR5 in CLL cells trafficking within the lymph nodes [16, 32, 50] and given that CXCR4 is associated with poor clinical outcome in CLL patients [10, 58, 59], we explored the clinical relevance of BCR-PKD-CXCR4/CXCR5 signaling pathway. Nearly all unmutated IGHV cases (35/36) exhibited responsiveness to BCR engagement in terms of CXCR4 downregulation. This correlation is not surprising regarding the cellular capacity to respond to BCR triggering and is consistent with disease progression as well as ultimately constant need of treatment in patients with unmutated IGHV [60, 61]. In contrast, in IGHV mutated cases, ability to down-regulate CXCR4 in response to BCR ligation identified two subgroups. In the first subgroup, 14 patients showed no or low CXCR4 decrease and none of them exhibited lymph node enlargement with a median follow up of 8.4 years; only 4 patients needed treatment and, for other reason than nodal progression. In the other subgroup of 22 IGHV mutated cases, with a stronger BCR/CXCR4 downregulation profile, a majority (64%) had tumor progression with lymphadenopathy, and 7 of them required treatment for nodal progression with a median follow up of 9.2 years. Altogether, our proposed model of BCR-mediated CXCR4 downregulation is in agreement with *in vivo* deuterium glucose labeling experiments showing that CXCR4^low^ peripheral cell fraction is representative of the proliferative pool of cells [62].

Our data support that the intensity of CXCR4 downregulation reflects the proportion of the BCR responsive subclone in a given patient. Indeed, we have shown previously that CXCR4 and CD62L downregulation occurred concomittanly upon BCR triggering [10] and we currently demonstrated that intensity of CXCR4 and CXCR5 downregulation took place in a similar subset of cells. PI3K-δ inhibitors allow CLL cells to exit from lymph nodes *in vivo* and represent an effective therapy. Interestingly, PKD inhibitors were shown to block pancreatic cancer growth in a xenograft mouse model [63]. Given the PKD implication in BCR-dependent CXCR4/CXCR5 internalization and CLL cell migration, targeting PKD could be an interesting alternative therapeutic option that would circumvent the PI3K-δ inhibitors side effects that target ΑΚΤ/mTor signaling pathways [64].

In conclusion, we identified a new PI3K-δ/PKD signaling pathway that leads to CXCR4/CXCR5 downregulation and is activated upon BCR engagement in CLL progressive cases (Figure 8). This work highlights the importance of BCR responsiveness towards CXCR4 downregulation, irrespective of the IGHV mutational status, on progression of tumor burden.

## MATERIALS AND METHODS

### CLL cell isolation and culture

CLL blood samples were obtained from untreated patients, after informed consent and validation by the local research ethics committee from the Avicenne Hospital (Bobigny, France), in accordance with the Declaration of Helsinki. CLL B cells were purified by negative selection using RosetteSep Human B Cell Enrichment Cocktail (StemCell Technologies, Grenoble, France). Purity (98,71% ± 1,41) was assessed as previously described [7]. CLL B cells were cultured fresh or after viable thawing in RPMI 1640 supplemented with 10% FBS, 100 U/mL penicillin, 100 μg/mL streptomycin, and 2 mM L-glutamine (PAA, Les Mureaux, France). For BCR stimulation, CLL B cells (4×10^6^ cells/well/12-well plate) were incubated with immobilized rabbit anti-IgM antibody (10 μg/mL; Jackson Immunoresearch, Montlucon, France) and incubated or not with Gö6976, Gö6983 (Calbiochem, Saint-Quentin-en-Yvellines, France), GF109203X, LY294002 (Sigma-Aldrich, Saint-Quentin-Fallavier, France), CAL101 (Selleckchem, Souffelweyersheim, France), CID755673 (Tocris Bioscience, Lille, France) or phorbol 12-myristate 13-acetate (PMA) (Cell Signaling Technology, Saint-Quentin-Fallavier, France). Human 293T and HepG2 cell lines were maintained in DMEM supplemented with 10% FBS.

### Flow cytometry analysis

CD5, CD19 and CXCR4 membrane expression levels were analyzed by flow cytometry (FACS-CANTO II; Becton Dickinson) after labeling with the indicated conjugated monoclonal antibodies (mAbs): PE-Cy7-anti-CD5, APC-Cy7-anti-CD19, APC-anti-CXCR4, PE-CXCR5 and FITC-CD62L or the respective control IgG isotype mAbs (BD Biosciences, Pont-de-Claix, France). Cell viability and plasma membrane CXCR4, CXCR5 and CD62L expression levels were determined in CD19^+^/CD5^+^ CLL cells. Data acquisition and analysis were performed using BD FACSDiva software. A threshold was arbitrarily set up case-by-case on untreated cells to include at least 90% of CXCR4^high^ or CXCR5^high^ or CD62L^high^ cells. BCR- and PMA-dependent CXCR4 or CXCR5 or CD62L membrane decreases were calculated as follows:

100 - ((% CXCR4^high^/CXCR5^high^/CD62L^high^ after IgM stimulation ×100) / (% CXCR4^high^/CXCR5^high^/CD62L^high^ before IgM stimulation)).

### Immunoblot analysis

After BCR stimulation or PMA treatment, total B cell contents were extracted using 1% Nonidet P-40 lysis buffer containing 50 mM Tris-HCl pH 7.5, 150 mM NaCl and 0.5 mM EDTA, supplemented with proteases inhibitors (Sigma-Aldrich). Proteins were separated on SDS-PAGE and analysed by western blotting with the appropriate antibodies: anti-phospho-Erk1/2, anti-PKD1/2, anti-phospho-Ser^744/748^ PKD, anti-phospho- Ser^916^ PKD, anti-HSP90 (Cell Signaling Technology), anti-PKD2 (Millipore, Molsheim, France), anti-PKD3 (Bethyl Laboratories Inc, Souffelweyersheim, France), anti-CXCR4 (Abcam, Paris, France), anti-phospho-Ser^339^ CXCR4 (Abcam), anti-phospho-Ser^324/325^ CXCR4 (ECM Biosciences, Souffelweyersheim, France) and anti-α-Tubulin (Sigma-Aldrich). Detection was achieved using chemi-luminescence (ECL, GE Heathcare, Velizy-Villacoublay, France) and visualized using the ChemiDoc MP Imaging System (BioRad, Marnes-la-Coquette, France).

### RNA isolation, reverse transcription and Q-PCR

Total RNAs were isolated from cells using Trizol reagent (Invitrogen, Saint Aubin, France), purified (QIAGEN, Courtaboeuf, France) and quantified by spectrophotometry. Total RNAs (1 μg) were retro-transcribed using MMLV reverse transcriptase. Quantitative real-time PCR (Q-PCR) was performed using a 7500 SDS Thermal Cycler (Applied Biosystems, Saint Aubin, France). Complementary DNAs, 3.2 μM gene-specific sense and anti-sense primers, as well as specific FAM-MGB probes (Applied Biosystems) (Supplementary Table S2) and PCR Master Mix were mixed in a 25 μL volume. The reactions were performed as follow: 40 cycles at 98°C for 15 seconds and 58°C for 60 seconds. Each experiment was repeated in duplicates. Cyclophilin was used as internal gene control. Relative quantification of each PKD gene expression was determined using the Δt method and values are expressed as 2^Δt^.

### Chemotaxis assay

Chemotaxis assays were performed as previously described [10]. Briefly, CLL cells were stimulated or not with coated anti-IgM for 24 hours and treated or not with CID755673. For each condition, cells were transferred into an upper chamber of a Transwell culture insert (Corning Costar, Illkirch, France), which was moved into a well containing RPMI supplemented with CXCL12 (100 ng/mL). After 8 hours at 37°C, non-migrated cells in the upper chambers and transmigrated cells in lower chambers were collected and counted. All assays were performed in duplicate.

### Data analysis and statistics

Data and statistical analyses were performed using the GraphPad program (Prism Version 6, France).

## SUPPLEMENTARY FIGURES AND TABLES


