# Supplementary material for: Protein kinase D-dependent CXCR4 down-regulation upon BCR triggering is linked to lymphadenopathy in chronic lymphocytic leukaemia
Source: Oncotarget. 2016 Apr 26;7(27):41031–46. doi: 10.18632/oncotarget.9031 (PMC5173040; doi:10.18632/oncotarget.9031)
Supplement: Supplementary file 1 [file oncotarget-07-41031-s001.pdf]

Protein kinase D-dependent CXCR4 down-regulation upon BCR triggering is linked to lymphadenopathy in chronic lymphocytic leukaemia

Supplementary Material

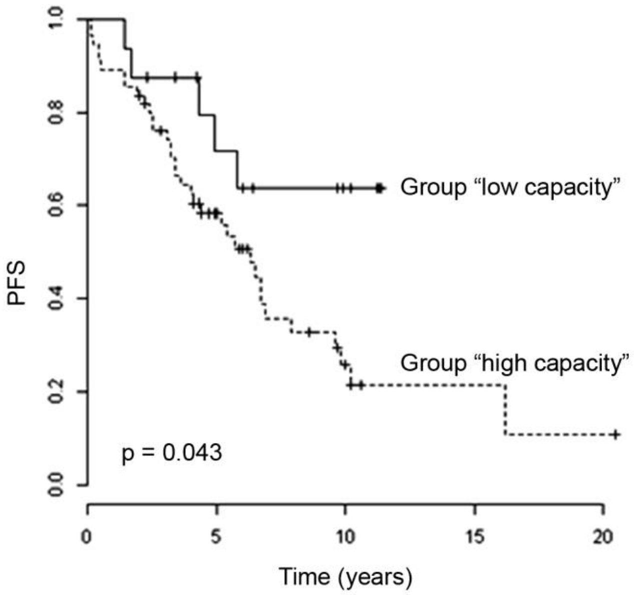

Supplementary figure S1**. BCR-induced CXCR4 decrease is associated with a risk of disease progression**. Progression free survival (PFS) curves (Kaplan-Meier) according to BCR-induced CXCR4 decrease. PFS was measured from diagnosis to first line treatment, death or last follow-up. Group “low capacity” (n=15): CXCR4 decrease ≤ 5%; Group “high capacity” (n=58): CXCR4 decrease > 5%. *P* values were calculated using log-rank test.

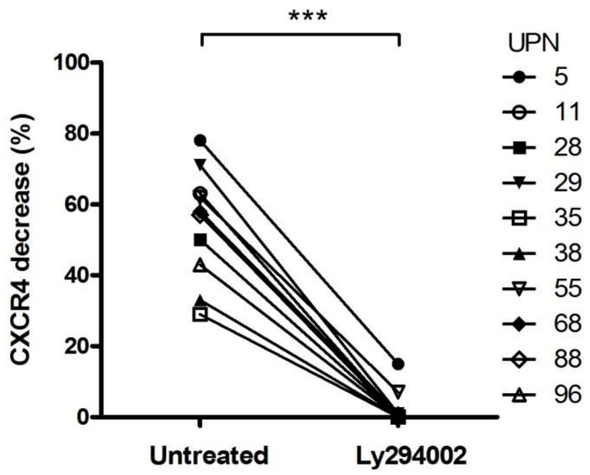

Supplementary Figure S2. **PI3K inhibition on BCR-induced CXCR4 down-regulation.** CLL B cells were treated with 20 µM Ly294002 or left untreated and stimulated with anti-IgM for 24 hours. Flow cytometry analyses were processed on $CD19^{+}/CXCR4^{+}$ cells and the percentage of CXCR4 decrease were calculated and graphed.

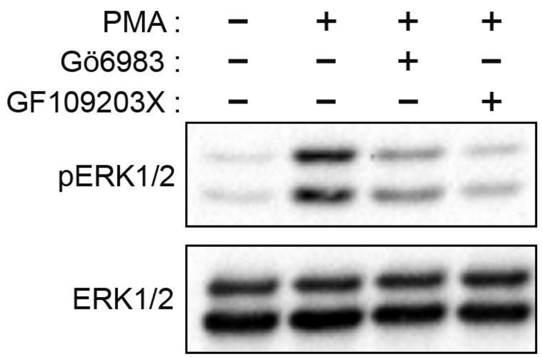

Supplementary Figure S3. **Gö6983 and GF109203X inhibitors blocked PMA-dependent ERK1/2 phosphorylation**. CLL cells (UPN 88) were stimulated for 15 min (+) or not (-) with PMA and/or treated (+) or not (-) with Gö6983 or GF109203X. Phospho-ERK1/2 and ERK1/2 levels were determined by western blot using the indicated antibodies.

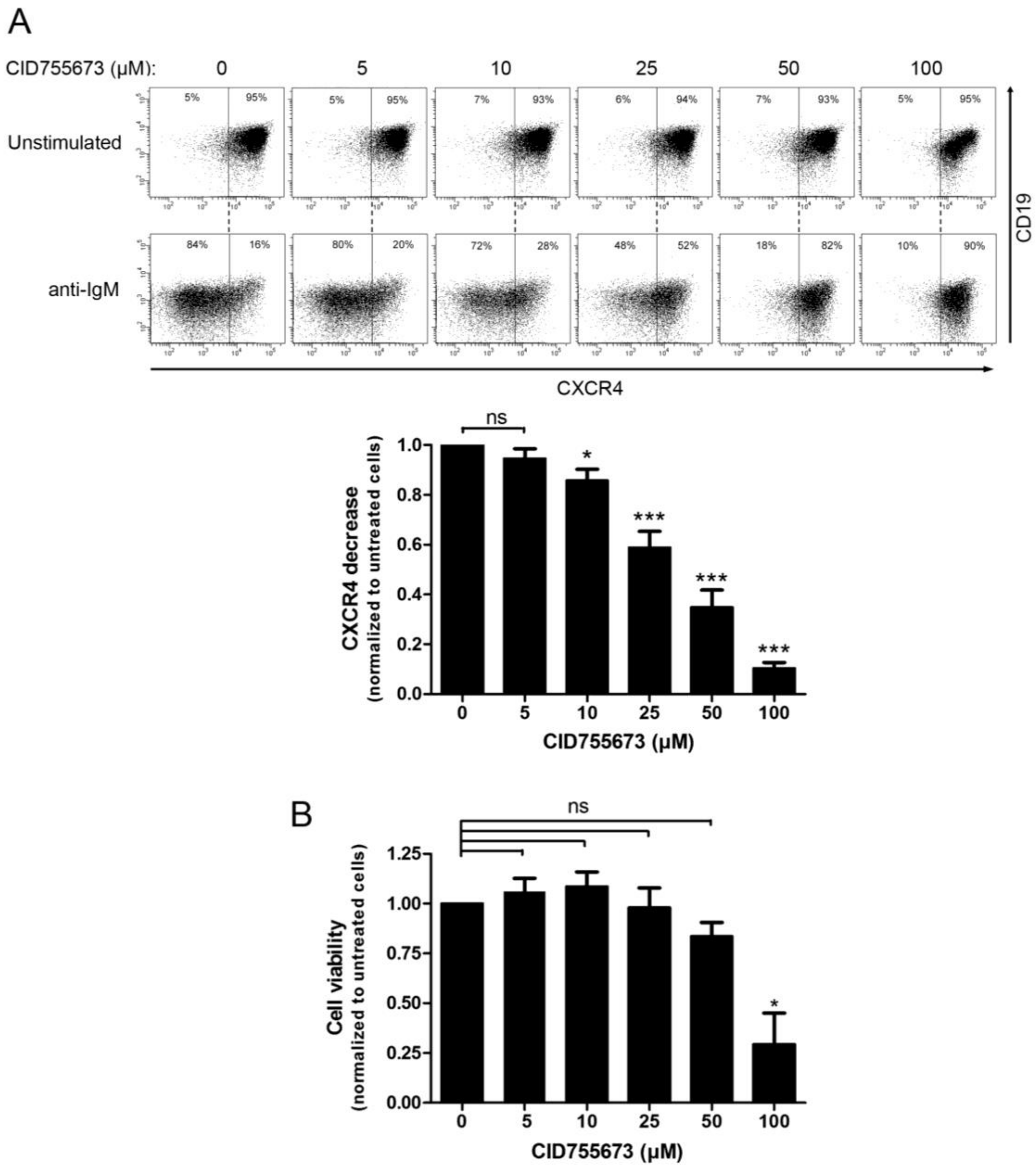

Supplementary Figure S4. **Optimisation of the CID755673 dose-response in CLL B cells.** CLL B cells were treated with the indicated CID755673 concentrations and stimulated (anti-IgM) or not (Unstimulated) for 24 hours. (A) Flow cytometry analysis of cell surface CXCR4 and CD19 expressions from a representative CLL sample (UPN 11) is presented. CXCR4 decrease upon anti-IgM stimulation from 9 CLL samples (UPN 11-98-99-105-113-117-121-122-123) was graphed (except for 100 µM CID, n=3, UPN 11-122-123); * $p<0.05$, *** $p<0.0001$. (B) CLL B cells were treated without or with 5 µM to 100 µM CID755673 and viability was assessed by flow cytometry and graphed; n=8 (UPN 11-46-98-105-114-117-122-123) for 0 to 50 µM CID755673 and n=4 (UPN 11-105-122-123) for 100 µM; * $p<0.05$; ns: not significant. Optimal concentration of 50 µM CID755673 was chosen for the study.

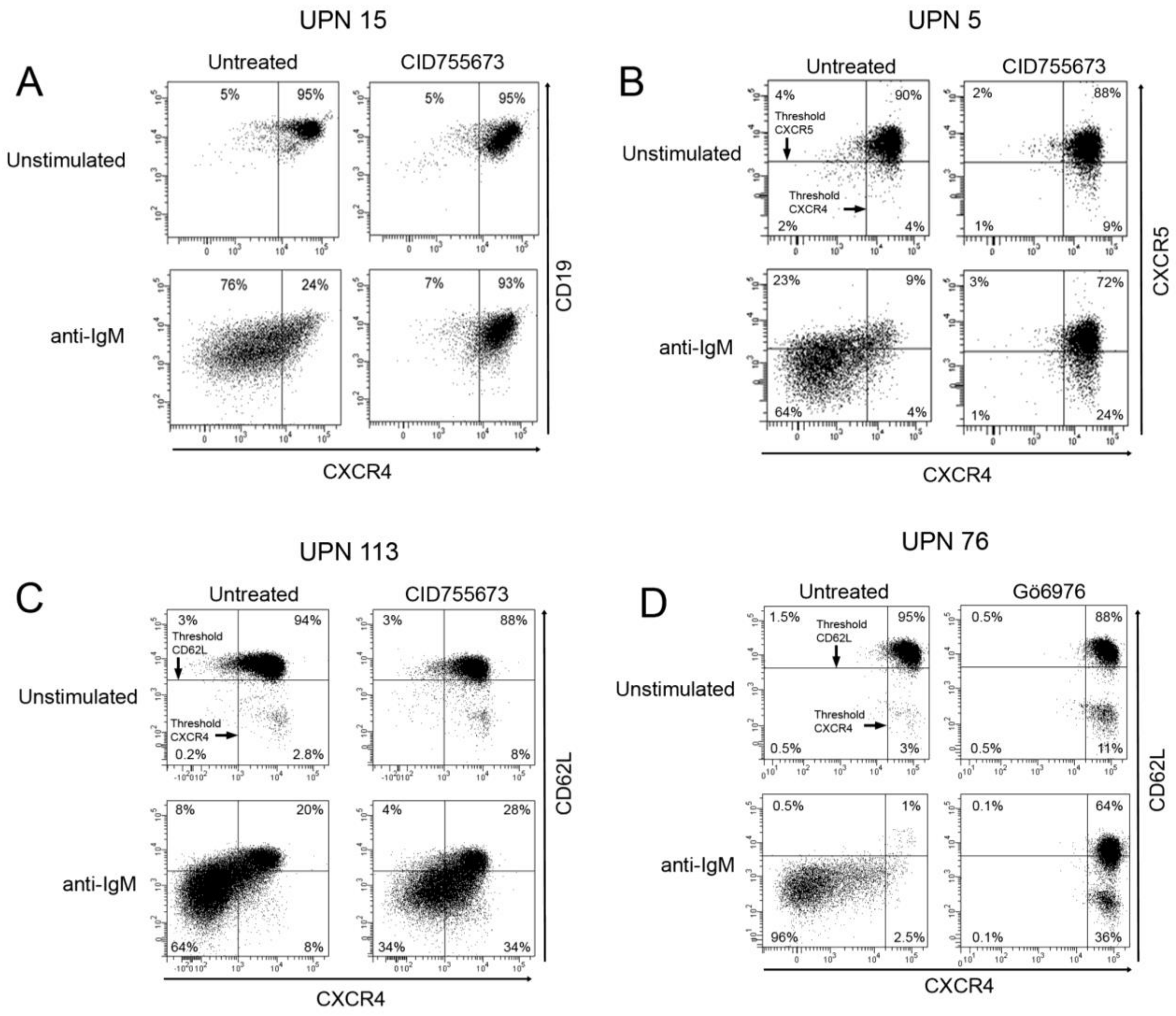

Supplementary Figure S5. **CID755673 inhibits BCR-mediated-CXCR4 and -CXCR5 internalization but not -CD62L down-regulation**. CLL B cells were left untreated or either treated with CID755673 (A, B and C) or Gö6976 (D) and stimulated (anti-IgM) or not (Unstimulated) for 24 hours. After gating $CD19^+/CD5^+$ cells, representative flow cytometry dot plots are presented showing membrane expressions of CXCR4 *vs* CD19 (A), CXCR4 *vs* CXCR5 (B), CXCR4 *vs* CD62L (C and D). Horizontal bars are the thresholds of CXCR5 (B) or CD62L (C and D) whereas the vertical one is the threshold of CXCR4 (A, B, C and D).

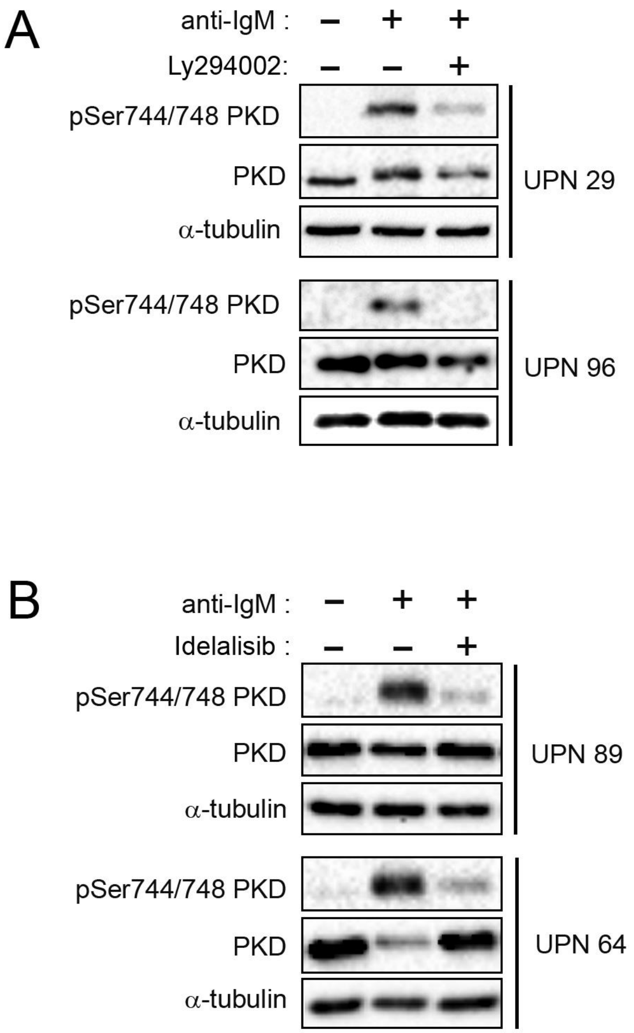

Supplementary Figure S6. **PI3K inhibitors blocked BCR-induced PKD-Ser$^{744/748}$ phosphorylation**. CLL cells were BCR-stimulated (+) or not (-) for 30 minutes in the presence (+) or not (-) of either 20 µM Ly294002 (A) or 50 µM Idelalisib (B). Western blot analyses were performed with the indicated antibodies and α-tubulin was used as a loading control.

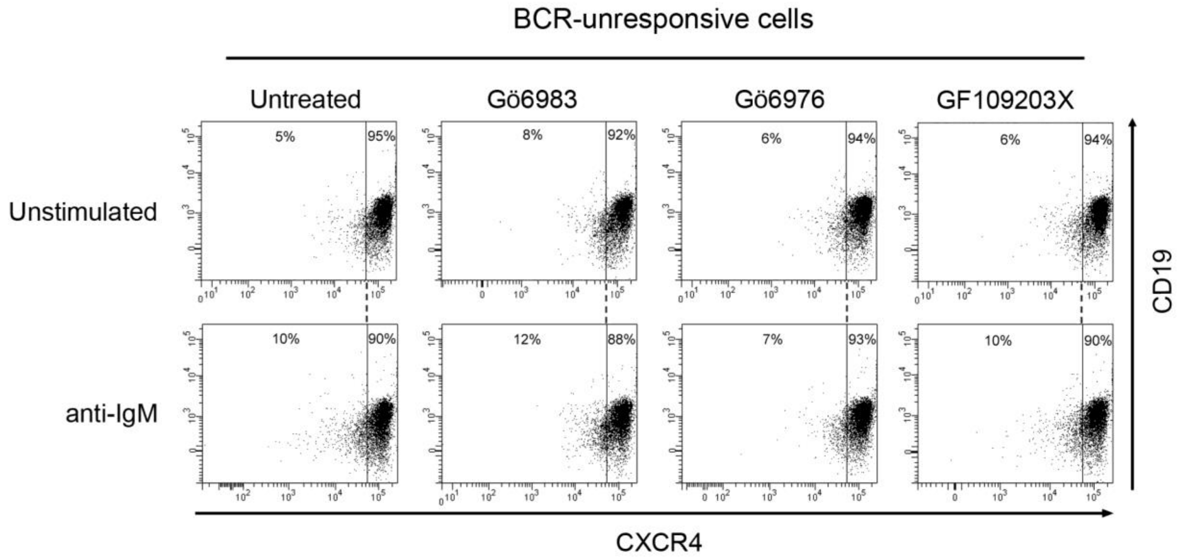

Supplementary Figure S7**. PKC/PKD inhibitory treatments of BCR-unresponsive cells did not alter plasma membrane CXCR4 levels**. After flow cytometry analysis, representative dot plots of cell surface CXCR4 and CD19 expressions are shown in a BCR-unresponsive cell sample (UPN 47) stimulated (anti-IgM) or not (unstimulated) and treated (Gö6983 (1 µM), Gö6976 (1 µM) and GF109203X (1 µM)) or not (untreated) with the indicated PKC/PKD inhibitors.

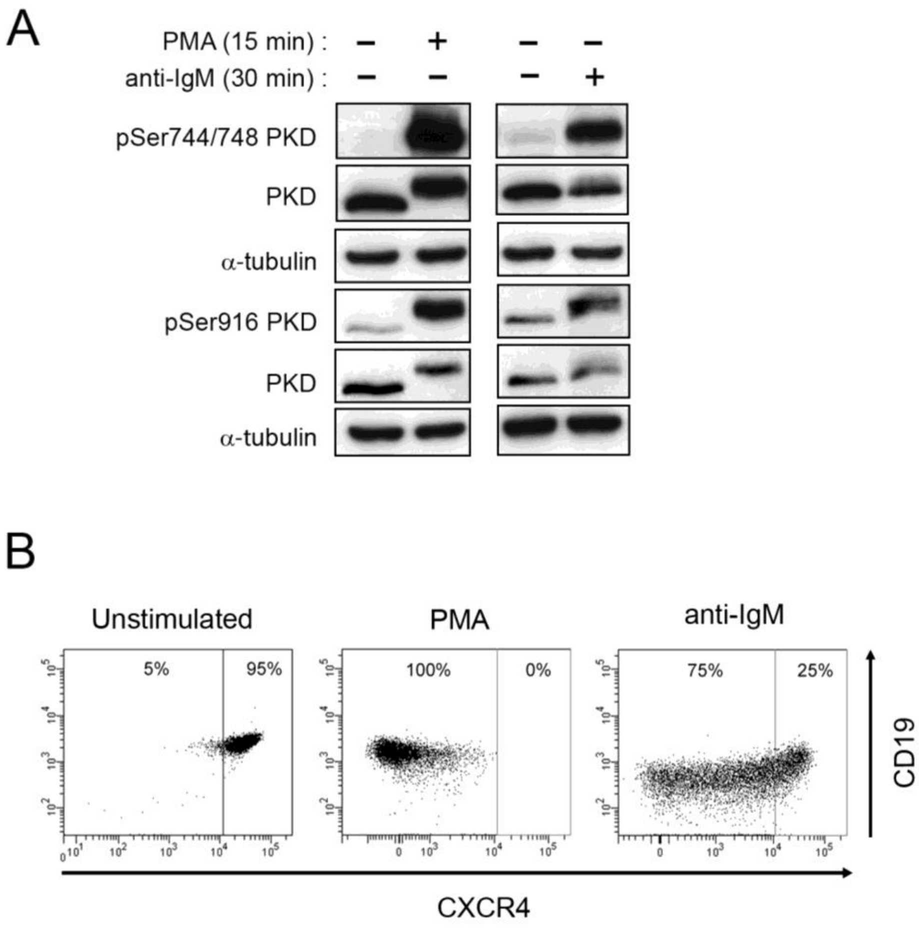

Supplementary Figure S8. **PMA treatment induced PKD phosphorylation increase and led to CXCR4 down-regulation in BCR-responsive CLL cells**. BCR-responsive CLL cells (UPN 7) were stimulated for the indicated time (A) or 24 hours (B) with PMA (200 nM) or anti-IgM antibodies. (A) Protein extracts were analyzed by western blotting with anti-pSer$^{744/748}$ PKD, -pSer^{916} PKD, -PKD or -α-tubulin antibodies. (B) CXCR4 and CD19 membrane expressions were determined by flow cytometry.

.

Supplementary Table S1: Biological parameters of 73 CLL cases (UPN) and correlation of BCR-induced CXCR4 decrease in relation to IGHV mutational status, BCR-induced cell survival and lymphadenopathy.

UPN	Gender[1]	BCR-induced CXCR4 decrease (%)	IGHV mutational status[2]	BCR-induced cell survival[3]	Lymph-adenopathy[4]
74	M	98	M	ND	Yes
76	M	96	UM	Pos	Yes
1	F	86	M	Pos	Yes
5	M	86	UM	Pos	Yes
18	M	85	M	Pos	Yes
120	M	81	UM	Pos	Yes
15	M	75	M	Pos	Yes
123	M	74	UM	Pos	No
7	M	74	M	Neg	Yes
118	F	73	UM	Pos	Yes
29	M	71	UM	Pos	Yes
113	M	71	M	Pos	Yes
99	M	70	UM	Pos	Yes
67	M	70	UM	Neg	Yes
32	F	66	UM	Pos	No
122	F	64	UM	Neg	Yes
11	F	63	UM	Pos	Yes
55	M	62	M	Neg	No
68	F	58	UM	Pos	No
12	M	58	M	Neg	Yes
88	F	57	UM	Pos	Yes
14	F	57	M	Pos	Yes
112	M	53	UM	Pos	No
59	M	52	UM	Pos	Yes
19	M	52	M	Neg	Yes
108	F	51	M	Pos	No
75	M	51	UM	ND	Yes
28	M	50	UM	Pos	Yes
100	M	49	M	ND	No
91	M	49	UM	Pos	Yes
36	M	45	M	Pos	Yes
96	M	43	UM	Pos	Yes
86	M	42	UM	Pos	Yes
48	M	42	UM	ND	No
117	M	41	UM	Pos	Yes
49	M	41	M	Neg	No
89	M	36	M	Pos	Yes
114	F	35	UM	Neg	No
98	F	35	M	Neg	No
64	F	34	UM	Pos	Yes
13	F	34	UM	Neg	Yes
101	M	33	M	Neg	Yes

38	M	33	UM	Neg	Yes
72	M	32	UM	Pos	No
42	M	31	UM	Neg	Yes
35	M	29	UM	Pos	Yes
107	F	28	M	Neg	No
57	M	23	UM	Neg	Yes
105	M	21	M	Neg	No
46	M	21	M	Neg	Yes
102	F	20	UM	Pos	No
71	M	20	UM	Pos	Yes
81	F	16	UM	Neg	Yes
53	F	16	UM	Pos	ND
121	F	14	UM	Pos	Yes
97	F	13	M	Neg	No
82	M	10	M	Pos	Yes
103	F	7	UM	Neg	Yes
93	F	5	M	Neg	No
84	M	5	M	Neg	No
83	M	5	M	Neg	No
63	M	5	M	Neg	No
47	M	5	M	Neg	No
110	M	3	UM	Pos	Yes
58	F	3	M	Neg	No
106	M	1	M	Neg	No
119	M	0	M	Neg	No
115	M	0	M	Pos	No
104	F	0	M	Pos	No
92	M	0	M	Neg	No
70	M	0	M	Neg	No
50	M	0	M	Neg	No
4	F	0	M	ND	No

Note: shaded boxes indicate unfavourable CLL factors.

[1] M= Male; F= Female

[2] UM= unmutated IGHV and M= mutated IGHV; Comparison of % BCR-induced CXCR4 downregulation between UM (46.5 ± 3.9; n=37) and M groups (31.3 ± 5.1; n=36) was significant (p= 0.0197) as previously described (Vlad A *et al*, Cancer Res 2009).

[3] BCR-induced metabolic activity (MTS); positive (Pos) MTS (> 25%) and negative (Neg) MTS (< 25%) responses (Deglesne PA *et al.*, Cancer Res 2006 and Le Roy C. *et al.,* Blood 2012). Comparison of % BCR-induced CXCR4 downregulation between Pos (49.35 ± 4.4; n=37) and Neg MTS groups (25.16 ± 4.15; n=31) was significant (p= 0.0002).

[4] Presence (Yes) or not (No) of lymph node enlargement in CLL patients.

ND: not determined

Supplementary Table S2. PKD primers used for real-time PCR in CLL and normal B cells.

Target	Sense primer (5'-3')	Reverse primer (5'-3')	FAM-MGB probe (5'-3')	Amplicon size
PKD1	ctgcctggtgtgcctcagtatat	aagagcactaggtcttgcacagcta	aaactcaagacaatgcac	76 bp
PKD2	ctccacagcggttcttcaca	catccagtttgggcaggaa	ccagcaatgaactgttc	74 bp
PKD3	gctgagttgtatgtctttgcatagc	atggtgtcagtcagaacatgcat	ccactctgctgctaaa	66 bp
